# Mobile Mechatronic/Robotic Orthotic Devices to Assist–Rehabilitate Neuromotor Impairments in the Upper Limb: A Systematic and Synthetic Review

**DOI:** 10.3389/fnins.2018.00577

**Published:** 2018-09-05

**Authors:** Gelu Onose, Nirvana Popescu, Constantin Munteanu, Vlad Ciobanu, Corina Sporea, Marian-Daniel Mirea, Cristina Daia, Ioana Andone, Aura Spînu, Andrada Mirea

**Affiliations:** ^1^Department of Physical and Rehabilitation Medicine, Carol Davila University of Medicine and Pharmacy, Bucharest, Romania; ^2^Emergency Clinical Hospital Bagdasar Arseni, Bucharest, Romania; ^3^Computer Science Department, Politehnica University of Bucharest, Bucharest, Romania; ^4^National Teaching Center for Neuro-Psyhomotor Rehabilitation in Children N. Robanescu, Bucharest, Romania

**Keywords:** upper limb rehabilitation, robotic exoskeletons, mobile robotic orthotic devices, mechatronic wearable orthoses, systematic and synthetic review

## Abstract

This paper overviews the state-of-the-art in upper limb robot-supported approaches, focusing on advancements in the related mechatronic devices for the patients' rehabilitation and/or assistance. Dedicated to the technical, comprehensively methodological and global effectiveness and improvement in this inter-disciplinary field of research, it includes information beyond the therapy administrated in clinical settings-but with no diminished safety requirements. Our systematic review, based on PRISMA guidelines, searched articles published between January 2001 and November 2017 from the following databases: Cochrane, Medline/PubMed, PMC, Elsevier, PEDro, and ISI Web of Knowledge/Science. Then we have applied a new innovative PEDro-inspired technique to classify the relevant articles. The article focuses on the main indications, current technologies, categories of intervention and outcome assessment modalities. It includes also, in tabular form, the main characteristics of the most relevant mobile (wearable and/or portable) mechatronic/robotic orthoses/exoskeletons prototype devices used to assist-rehabilitate neuromotor impairments in the upper limb.

## 1. Introduction–general perspective and main rationales

What differentiates human beings from animals is the superior psycho-cognitive activity, including the coordinated/complex, workable, actions of its highly correlated physical effecter: the upper limb, and especially the hand—as basis of our creative and modeler/draftsman kind interactions with the environment. This profound and subtle reality has been conceptualized during history by great thinkers, such as Aristotel (2005), Descartes, Newton and Kant (Lundborg, 2014).

Accordingly, finding solutions that address rehabilitation and/or functional assistance of neuromotor impairments at this level would have a remarkable positive impact: for the beneficiaries' quality of life (Frisoli et al., 2016) and from a socio-economical perspective, as well. The latter corresponds to the temporary regain/re-insertion of the productive resources lost because of the disabilities in their upper limbs. Moreover, it is to be considered, within the general context/trend of offering a reliable alternative for prolonged hospitalizations, the need for top of the range assistive/rehabilitative orthotic mobile devices. These should be capable to provide safe and of continuity rehabilitation (Loureiro et al., 2011) and/or functional assistance for the above mentioned topography, too, of neuromotor deficits including in the patient's daily life context. Such endeavors are often necessary on long term, mainly imposed, in the morbidity domain we approach, by the required duration of neuroplasticity to install/act (Muresanu et al., 2012; Basteris et al., 2014; Xiao et al., 2014; Proietti et al., 2016; Mazzoleni et al., 2017), to be (re)settled in adequate engrams for the function(s) aimed at restoring, and/or of peripheral nerves' re-growth (Guyton and Hall, 2006).

The necessity for such devices that can operate without fatigue in both clinical settings, at home and in the community is growing high (Stewart et al., 2017). This is despite of the fact that people with upper limb pathology—who do not necessarily suffer from functional issues in the lower limbs—can commonly reach clinical units (in order to receive ambulatory rehabilitative specific procedures). Another aspect is that, at the moment, there is already a “shortage” of professionals handy to deliver domiciliary physiotherapy/rehabilitation and nursing, for persons with physical impairments. This is a worrying situation, especially as it is foreseen to become more and more frequent in the years to come (Maciejasz et al., 2014).

An important related development direction consists of consolidating their wearable profile. This practically entails—subsumed to a rightful beneficiary's desire: “several hours” per day of working performance (Allotta et al., 2015)—availability for autonomous powered duty (as for easily/rapidly rechargeable facilities, too) and respectively comfortable bearing by the consumer in the daily life (Giberti et al., 2014), limitation of encumbrances, lightweight (Rocon et al., 2007; Martinez et al., 2008; Song et al., 2013, 2014; Chen et al., 2014; Giberti et al., 2014; Andrikopoulos et al., 2015; Allotta et al., 2015; Polygerinos et al., 2015; Guo et al., 2016; Nycz et al., 2016; Alavi et al., 2017; Stewart et al., 2017) and modularity (Lo et al., 2010; Pearce et al., 2012; Noveanu et al., 2013; Xiao et al., 2014; Nycz et al., 2016) and/or, in some cases, “reconfigurability” (Maciejasz et al., 2014).

Considering all the necessary technical assets for such advanced devices to be mobile (Kiguchi et al., 2008a; Lee, 2014; Nycz et al., 2016), thereby available for individual more extended use, an additional, non-technical, but derivative and decisive condition is, as well, mandatory: their cost-effectiveness (Noveanu et al., 2013).

We consider it only appropriate to iterate here a summarized idea of a previous work of ours (Onose et al., 2016) that currently there is still no such thing as an optimal, fully functional assistive-rehabilitative device (in the common sense of the term). This regards mainly: don/doff issues (Nimawat and Jailiya, 2015)–for severely disabled potential beneficiaries–, psychological acceptance (of self image/esteem kind, referring to the ensemble look of the consumer: enough miniaturization and cosmetics– thus either reaching a satisfactory clothes-like aspect or even becoming as thin as to evolve to underwear dimensions), extended power autonomy, easy and fast set-up-for professionals (Dijkers et al., 1991). Another important feature for the customers/their kin is the appropriateness for long time duty in various real life situations. One should consider also the consistent related safety, producing very low/practically imperceptible noise when in service and truly affordable/cost effective.

## 2. Methods

Despite the rigorous selection filter-classification criteria based methodology of the papers we have reviewed, some data referring to the subject matter approached, might still be overlooked. At the same time, on one hand, not all the selected articles contained aspects needing to be elicited and expressly quoted. On the other, for clarifying different notions (collateral but nevertheless, important for this paper) we also used Supplementary—to the portfolio gathered availing the below mentioned combinations of keywords—bibliographic resources.

According to the afore exposed rationale, our research considered publications from January 2001 to November 2017 from the following databases: Cochrane^1^, Medline/PubMed^2^, PMC^3^, Elsevier^4^, PEDro^5^, ISI Web of Knowledge/Science^6^.

**Figure 1:**
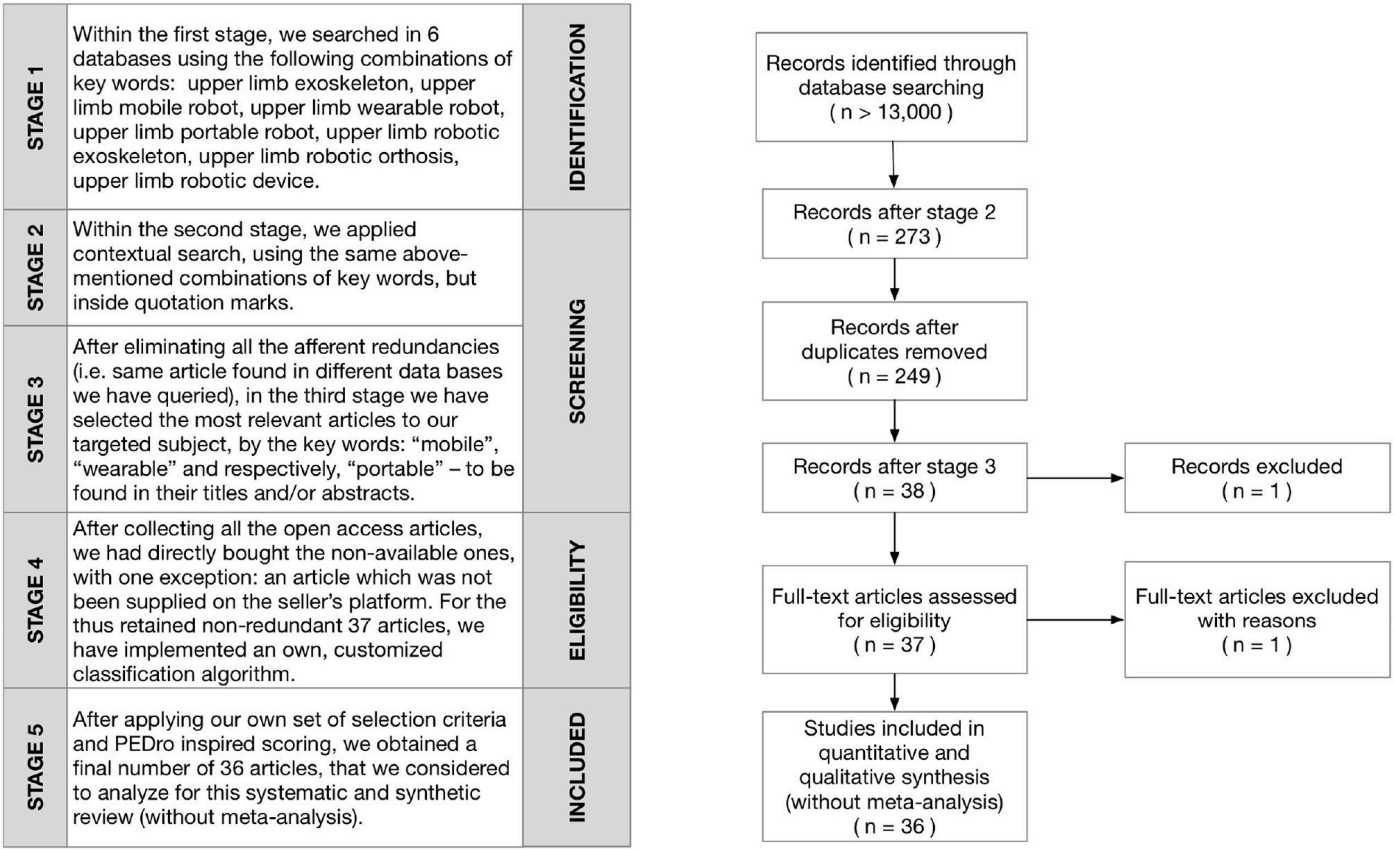
PRISMA adapted flow diagram of the method used for the articles' systematic selection.

Our search has been conducted on five stages, described by a Preferred Reporting Items for Systematic Reviews and Meta-Analyses (PRISMA)^7^ adapted flow diagram, but without the final meta-analysis stage (see Figure 1). Within the first stage, using the following combinations of keywords: upper limb exoskeleton, upper limb mobile robot, upper limb wearable robot, upper limb portable robot, upper limb robotic exoskeleton, upper limb robotic orthosis, upper limb robotic device, it resulted, as expected—even if overlapping—a huge amount of articles: (more than 13,000-details in Table 1).

**Table 1:** Step I: numerical search results.

**Keywords**	**Cochrane**	**PubMed**	**PMC**	**Elsevier**	**PEDro**	**ISI**	**Total**
*upper limb exoskeleton*	585	289	807	18	3	674	2,376
*upper limb mobile robot*	763	12	368	20	0	33	1,196
*upper limb wearable robot*	599	41	349	15	1	122	1,127
*upper limb portable robot*	611	13	319	3	0	24	970
*upper limb robotic exoskeleton*	599	210	682	17	1	314	1,823
*upper limb robotic orthosis*	606	94	632	20	3	66	1,421
*upper limb robotic device*	964	638	2,107	90	12	495	4,306
Total	4,727	1,297	5,264	183	20	1,728	13,219

Therefore, we still had to achieve a mandatory refinement: within the second stage, we applied contextual search, using the same above mentioned combinations of keywords, but inside quotation marks. It then resulted in over 270 articles (details in Table 2).

**Table 2:** Step II: numerical search results.

**Keywords**	**Cochrane**	**PubMed**	**PMC**	**Elsevier**	**PEDro**	**ISI**	**Total**
“*upper limb exoskeleton”*	0	33	63	3	1	155	255
“*upper limb mobile robot”*	0	0	0	0	0	0	0
“*upper limb wearable robot”*	0	0	0	0	0	1	1
“*upper limb portable robot”*	0	0	0	0	0	0	0
“*upper limb robotic exoskeleton”*	0	3	1	0	0	12	16
‘*upper limb robotic orthosis’*	0	0	0	0	0	0	1
“*upper limb robotic device”*	0	0	0	0	0	0	0
Total	0	36	64	3	1	169	273

After eliminating all the inevitable redundancies (i.e., same article found in different queried data bases), in the next stage we have selected the most relevant articles to our targeted subject by the keywords: “mobile,” “wearable,” and respectively, “portable”—to be found in their titles and/or abstracts. This has reduced the number of results to: 3 articles for “mobile,” 29 for “wearable,” and 12 for “portable“ (but among them, 6 contained both, the “wearable” and “portable” keywords). After collecting all the respective open access articles, we had directly bought the non-available ones (with an exception: one article which was not available on the seller's platform) and then retained 37 non-redundant articles. On these we have implemented an own, customized, PEDro inspired, classification algorithm—described below.

In this purpose, we have previously considered connected literature knowledge from the Population/ Intervention/Comparison/Outcome/Time–PICOT (Fineout-Overholt et al., 2010; Riva et al., 2012)–and Study Type–PICOS (Methley et al., 2014)–, Feasible/Interesting/Novel/ Ethic/Relevant–FINER–(Farrugia et al., 2010) and Physiotherapy Evidence Database criteria—PEDro^8^—(Maher et al., 2003). We had in mind some essential/defining features probable to be found in the approached domain (specifically, in our selected articles): studies using prototypes, with single (24.32%), small (21.62%), or multiple (10.81%) case/s series (details and related references in the results section). Additionally, the studies of interest for our subject matter may involve different interventions, with mobile robotic orthotic devices, where randomization “concealed/blindness” criteria are difficult to be applied/(not always found), observations on healthy subjects (63.28%)—details and related references in the results section—various assessment scales and/or end-points, used.

Consequently, we have developed [based on a Delphi kind preliminary related endeavor: brainstorming between the authors—as being a multi-/inter-disciplinary staff team^9^ —(Verhagen et al., 1998)] an own customized panel of criteria, using an up to 10-points, PEDro-inspired, scoring for classifying the articles assessed details in Appendix 1 within the Supplementary Material.

The articles were selected only if they had been written in English. We only kept those articles that obtained at least 4 points (“fair”/“high” quality^10^), considering the following grading criteria (Q1–Q4 as shown in Appendix 1 within the Supplementary Material): published in a journal within the Institute for Scientific Information (ISI) Thomson Reuters^11^/International Data Bases (IDB), indexed; number of citations per year, pondered (see below) through the year of publication; number of human subjects included in the study (this criterion does not apply for review articles: for such papers there have been considered, following the same calculation formula, the other three criteria); the references' quality (no reference: 0 points; 1–10: 1 point; 11–20: 2 points; 21–30: 3 points; 31–40: 4 points, 41 and over: 5 points).

For each criterion, the maximum number of points possible to be obtained was 5. In order to keep the symmetry with the other criteria regarding the PEDro inspired scoring, we have chosen to classify the articles' quality within the criterion referring to databases, such as: 3 points if the respective article is rated in minimum one IDB and 5 points if it is ISI Thomson Reuters indexed.

As announced above, in order to quantify the citation quality of an article an own customized formula was used, that takes into consideration the number of citations per year, the maximum number of citations per year for all candidate articles, and also the year in which the article was published.

First, the number of citations per year is computed using Equation (1).

(1) $$\begin{array}{l} \begin{array}{c} CPY_{i}=\frac{TC_{i}}{2018-Y_{i}} \end{array} \end{array}$$

where *CPY* stands for the citations per year, *TC* is the total number of citations and *Y* is the year in which the article was published (for article *i*).

In order to normalize the scores of various articles that were published in different years, a bonus scheme was developed to ensure that the number of citations for newer articles weigh more than for older ones with the same number of citations. By using Equation (2), the absolute value of the reference quality of an article is computed:

(2) $$\begin{array}{l} \begin{array}{c} Q_{i}^{*}=\frac{5*CPY_{i}}{max{\;}_{j=1\ldots \;n}\left(CPY_{j}\right)}+\frac{6-min\left(2018-Y_{i},6\right)}{2} \end{array} \end{array}$$

where $Q_{i}^{*}$ represents the absolute value of the reference quality of article *i*.

Finally, all absolute values are limited to the interval [0 : 5] by using Equation 3.

(3) $$\begin{array}{l} \begin{array}{c} Q_{i}=\{\begin{array}{cc} \;Q_{i}^{*}, & Q_{i}^{*}<5\\ 5, & Q_{i}^{*}\geq 5 \end{array} \end{array} \end{array}$$

where *Q*_*i*_ is the final value of the reference quality of the evaluated article.

The article's total score is obtained as the average of each considered criterion calculated points multiplied with 2 (in order to range the maximal score up to 10). After applying this set of selection criteria, a final number of 36 articles remained. We analyzed them for this systematic review.

It is to be mentioned a sui generis situation in which, after the detailed analysis of all articles, one of them, with 6 points PEDro inspired score (Lin et al., 2013a) even if it matched the contextual search syntax “upper limb exoskeleton” within its combination of keywords, the content of the paper referred to an arm support mobile accessory—possibly just adjunct for mechatronic/robotic orthoses/exoskeletons—but without having such technical hallmark features: neither actuators nor sensors, but only mechanical passive balancing gravity components.

## 3. Outcomes of our systematic review

The most relevant mobile (wearable and/or portable) mechatronic/robotic orthoses/exoskeletons—to approach neuromotor impairments in the upper limb—prototype devices are detailed in Appendix 2 within the Supplementary Material. The equipment used in the literature we have revised is systematized in the respective table, also containing other such prototype devices met in the supplementary references (marked as *italic*).

**Figure 2:**
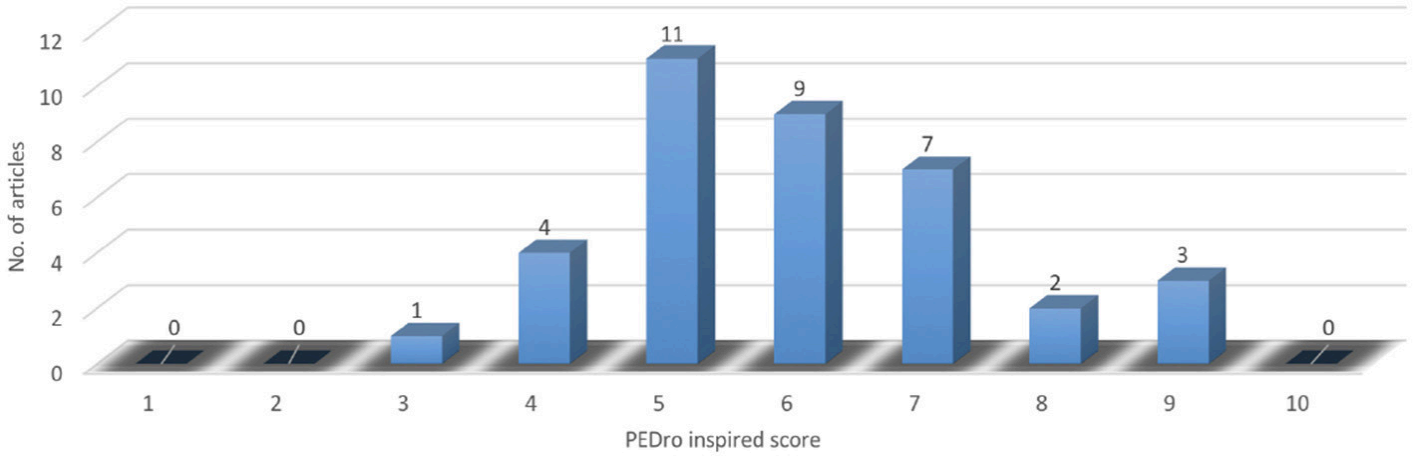
Articles' PEDro inspired score distribution.

**Figure 3:**
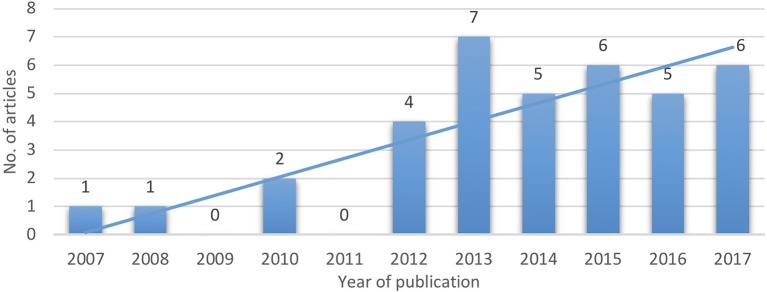
Selected articles' years distribution.

Concerning the score distribution among the analyzed articles (Figure 2), it is to be emphasized its nearly Gaussian pattern (Pearson asymmetry coefficient: 0.63), thus supporting the accuracy of the selection process we have done. This reflects, at the same time, the fact that the articles were ranked as of “fair” to “high” quality, Average: 5.95; Median: 6–similar to other works (Veerbeek et al., 2017)–Dispersion: 1.81; Standard deviation: 1.5; Coefficient of variation: 25.23%. Additionally, Figure 3 displays the ascending trend of the issues per year among the articles we have selected, which is consistent with the related opinions in literature (Lo, 2012; Maciejasz et al., 2014; Proietti et al., 2016; Veerbeek et al., 2017). It is to be noted the “boom” of such articles in 2013.

**Figure 4:**
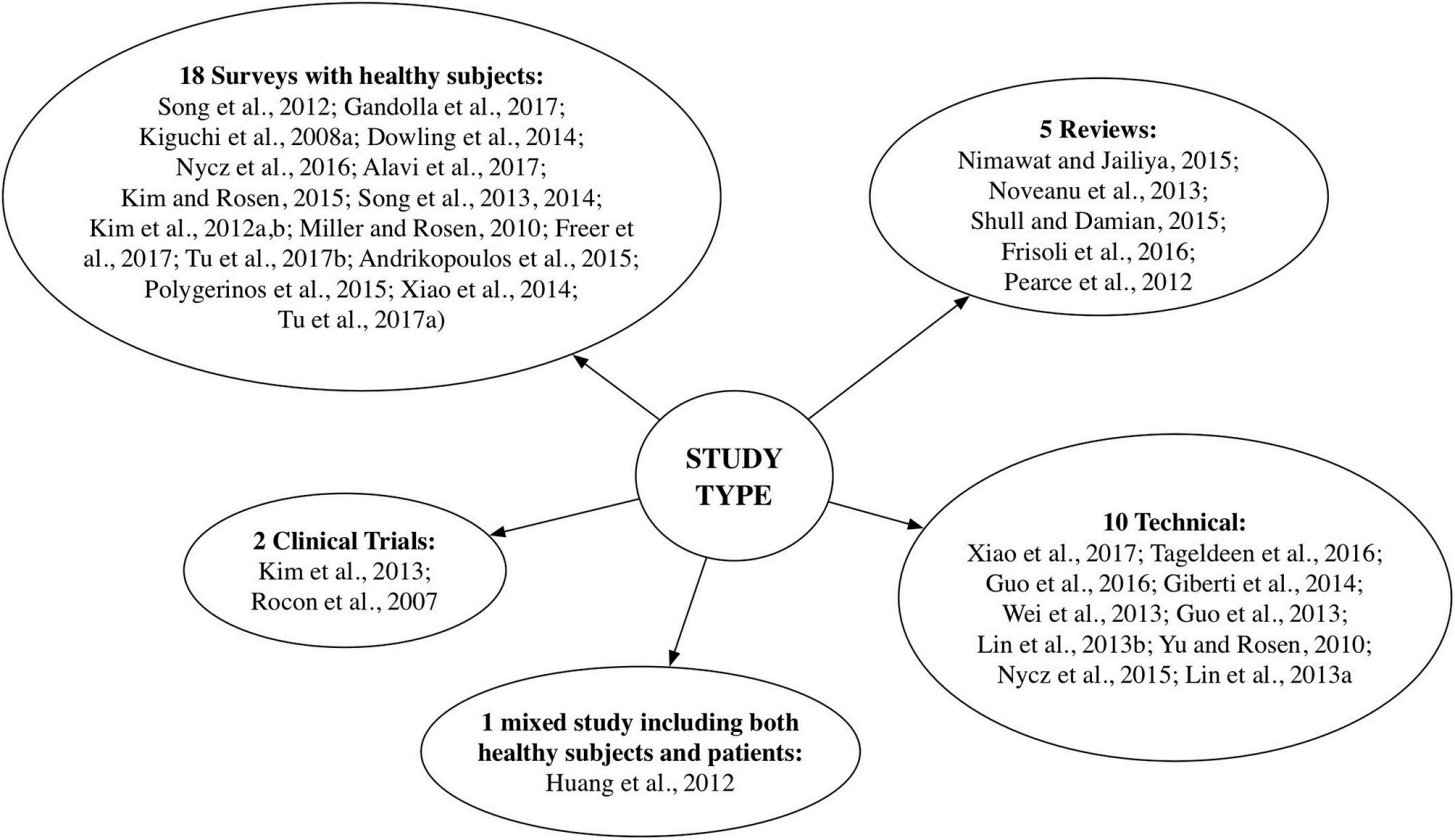
Taxonomy of the selected articles.

Taxonomically, we have sorted the selected and qualified articles into five categories as shown in Figure 4.

**Figure 5:**
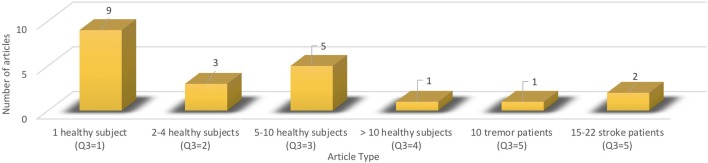
Distribution of the selected articles involving human subjects.

**Figure 6:**
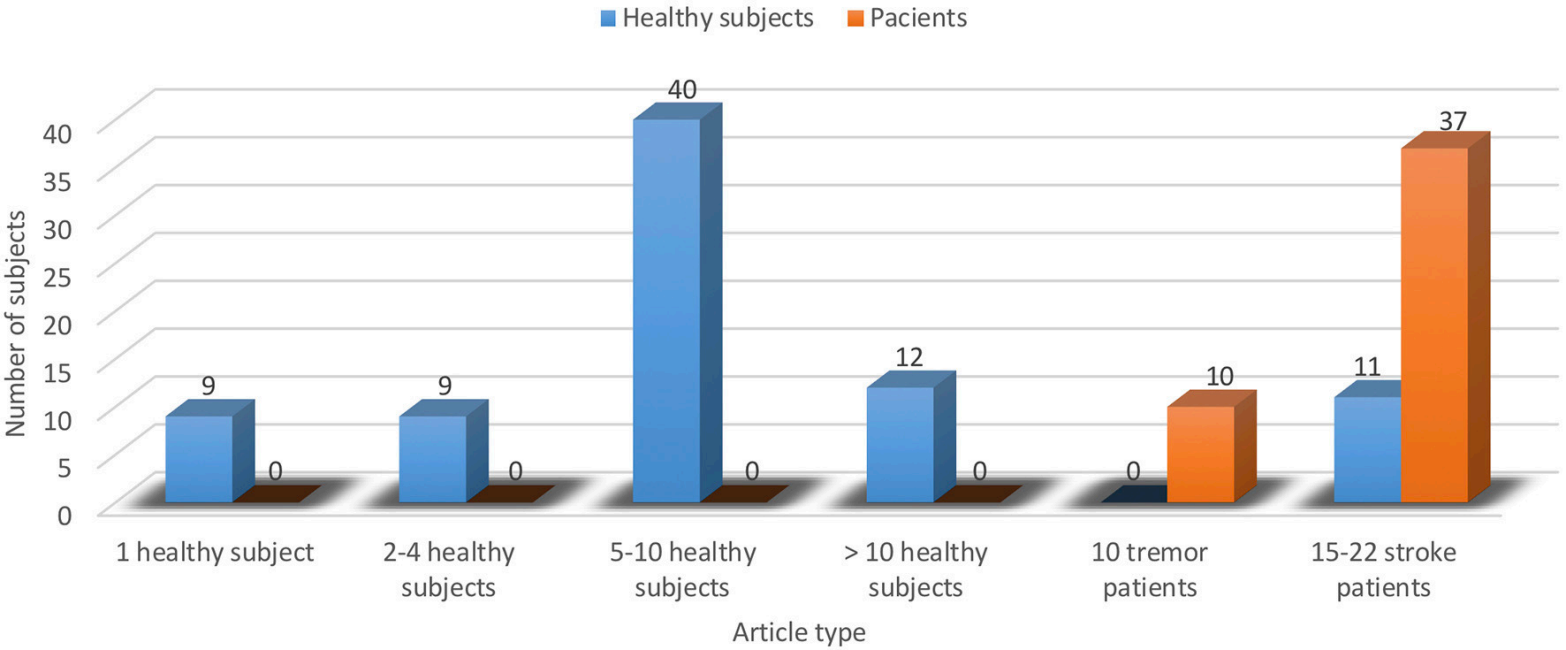
Distribution among the selected articles of enrolled human subjects.

As for the relevance, based on the number of cases assessed within trials evaluating the interventions done with considered mechatronic/robotic devices in upper limb neuromotor impairments, the situation determined in the analyzed articles is presented in Figures 5, 6. It may be observed that overall, there are no clinical studies with large database of cases, although in recent supplementary related literature, much larger samples can be found (Takahashi et al., 2016).

Specifically, the majority of the studies (18) assessed human-robotic device interactions with healthy individuals (totally 81 subjects) and only 3 articles included patients: 37 with stroke (15 Kim et al., 2013 and 22 Huang et al., 2012), and respectively, 10 with tremor (Rocon et al., 2007).

## 4. Discussion

An important finding regards a quite promising and actual development trend: tracking for feedback and training arm (shoulder, elbow, forearm) and hand, together, produced “greater improvement” than such endeavors done separately for the respective anatomic regions (Merians et al., 2009). The methods to achieve that require the use of, including haptic-based, virtual reality (VR) facilities (Huang et al., 2012; Guo et al., 2013; Lin et al., 2013b; Wei et al., 2013; Dowling et al., 2014; Song et al., 2014; Thielbar et al., 2014; Kim and Rosen, 2015; Shull and Damian, 2015; Grimm et al., 2016; Mazzoleni et al., 2017; Maris et al., 2018). This matches with the conceptual addition of, for instance, Brain Machine Interface (BMI) and/or neuromuscular electrical stimulation (NMES)/FES or transcranial Direct Current Stimulation (tDCS), respectively repetitive transcranial magnetic stimulation (rTMS), facilities usage, too. Furthermore and more recently: some multimodal/hybrid such advanced devices may also provide compensation of gravity load by the related exoskeleton (Yu and Rosen, 2010; Kim et al., 2013; Giberti et al., 2014; Song et al., 2014; Andrikopoulos et al., 2015; Gandolla et al., 2017; Tu et al., 2017a), in order to improve robotic neurorehabiliative/assistive interventions' outcomes, including in the upper limb (Grimm et al., 2016; Tu et al., 2017a; Mazzoleni et al., 2017; Stewart et al., 2017).

At the same time, it proves to be not only actual, but also in line with the ascending trend toward “all-in-one” and/or modular kind of designs (including) for the robotic/mechatronic assistive-rehabilitative orthoses/exoskeletons. This is to be foreseen as neuroscience and technology advance, and respectively, the consumers' expectations for comfort and effectiveness increase, over time, thus supporting our option to focus this systematic synthetic review on mobile advanced systems. This type of apparatus can be divided into two categories: portable and wearable. The latter include devices using fabric integrated within smooth but robustly fit (Polygerinos et al., 2015; Nycz et al., 2015; Rus and Tolley, 2015; Onose et al., 2016). One must consider, at the same time, cosmetics: more complex, but light weight (Rocon et al., 2007; Martinez et al., 2008; Song et al., 2013, 2014; Chen et al., 2014; Giberti et al., 2014; Andrikopoulos et al., 2015; Polygerinos et al., 2015; Guo et al., 2016; Nycz et al., 2016; Alavi et al., 2017) and, at least externally, garment–like structures.

Another quite recent-justified as being a key item that synthesizes, like a top of iceberg, many of the essential mechatronic determinants is the so-called “transparency” (Kim and Rosen, 2015; Proietti et al., 2016). This actually tends to improve the overall outcome, by being underpinned including on increased intuitiveness thus matching with the user's non invasively extracted EMG and/or EEG/BCI movement purposes (Onose et al., 2012). It also provides references and adjustments (and subsequent feed forwards) within a higher level of related abstractness. All these are subsumed to its artificial intelligence/“wisdom” of not acting when not necessary. Such aspect is required for fulfilling overall enhanced (thus being more and more bionic/biomimetic, too) expected performances of robotic exoskeletons, that address also upper limb neuromotor deficits. Therefore, transparency would be, at the same time, a valuable marker for the accuracy of task-oriented achieved results, which could be obtained following human-robotic interactions (Proietti et al., 2016).

More substantiation is needed for the related quest improvement, and it mainly refers to not enough trials on extended groups of patients, complete and clear description of the research methodologies used, statistical power and—based on clinical results—data processing, randomization, (lack of) control lots, “dropout rate” reporting etc. (Lo, 2012).

Because of the mandatory necessity for an (inevitable) multi-/inter-disciplinarity domain's profile, papers can be found in the literature, well documented but based on trials with different or not always best adequate design/statistical power and/or methods used to assess the outcomes obtained. Among these, some present positive or neutral, while others, partially negative findings on robotized physiatric approaches of upper limb neuromotor impairments.

Possible limitations, at least in some circumstances, for (more) valid/“generic” conclusions (Proietti et al., 2016) are represented by the—intrinsic, tightly connected—particularities of various such devices and also the originality/technical advancements/contributions they bring (resulting in differences—objectively—difficult to be rigorously compared). For instance: some authors document no spectacular (if any) benefits—regarding motor function gain and cost-effectiveness—compared to classical, therapists administered, corresponding endeavors (Lo et al., 2010). Others question the effectiveness in improving ADL (divergent, i.e., beneficial Mehrholz et al., 2015) and express conflicting opinions about effects on muscle (hiper)tone states (favorable Sale et al., 2014 vs. negative Veerbeek et al., 2017). The assistance-as-need approach (rather nuanced reticent Norouzi-Gheidari et al., 2012 vs. positive Stewart et al., 2017), required dosage (Norouzi-Gheidari et al., 2012; Pollock et al., 2014; Stewart et al., 2017; Veerbeek et al., 2017) is also debated for pathologic evolution stage (better for chronic Mazzoleni et al., 2017; Veerbeek et al., 2017 vs. subacute Sale et al., 2014).

Generally, one can find discriminating opinions among the efficacy of different control strategies applied and/or between true motor rehabilitation and contextual functional compensations. The latter brings an overall added value to the patient's interaction with the environment, but cannot be clearly/specifically attributed to a certain intervention (Kwakkel et al., 2008; Marchal-Crespo and Reinkensmeyer, 2009; Veerbeek et al., 2017). Even the cost-effectiveness of such interventions remains to be proven: on mid-longer-term (time frame surveyed 36 weeks) the “total costs” of: “robot therapy,” “intensive comparison therapy” and “usual care” are “comparable” (Wagner et al., 2011). However, to balance these opinions, there can also be found in literature a quite converse overall vision on this subject: “Robotic aided therapy has shown to be more effective than traditional physical therapy in providing high intensity of exercise, better movement controllability and measurement reliability, which makes robots ideal instruments…” that “can deliver training at a much higher dosage…” (Frisoli et al., 2016).

Technological progresses are theoretically prone, on one hand, to enhance related therapeutic effectiveness and on the other, to reduce afferent costs. There are interesting divergent assertions in this respect, too: more advanced such equipments—possibly being, conversely, more expensive. However, such advances do not necessarily improve effectively the main outcomes targeted: voluntary controlled motility, muscle power and overall functionality/ADL (Veerbeek et al., 2017). But they can still offer some advantages, such as better adherence to therapy through the psychological investment. This includes patient wishful thinking, provided by novelty and equipment complexity. Also, these devices are also capable of better assistive functions and/or longer—thus more intensive—rehabilitation (Norouzi-Gheidari et al., 2012), based on much more repetitive “motor learning” (Charles et al., 2005) and task/“goal-orientated,” movements (Grimm et al., 2016).

Generally, the main risks, aside from those consequent to human involvement (for the participant and/or generated by the person—skilled or not—who aids the user in the respective man-machine interaction), related to the use of robotic exoskeletons for medical interventions in the upper limb, are: “joint misalignment, skin damage, software malfunction leading to uncontrolled behaviors, electrical and fire hazard” (He et al., 2017). Risks subsequent to possible malfunctions in pneumatic or hydraulic power systems will be emphasized further.

A limitation of our article: the strict/rigorous selection methodology we have applied resulted in rather few overall clinical cases found in the trials afferent to the works we have analyzed.

### 4.1. Main indications

Generally, for the mechatronic orthotic devices/robotic exoskeletons—including those acting in the upper limb—there are four main applicability fields: “Military, Industry, Medical, First Responders”(Neugebauer, 2017).

**Figure 7:**
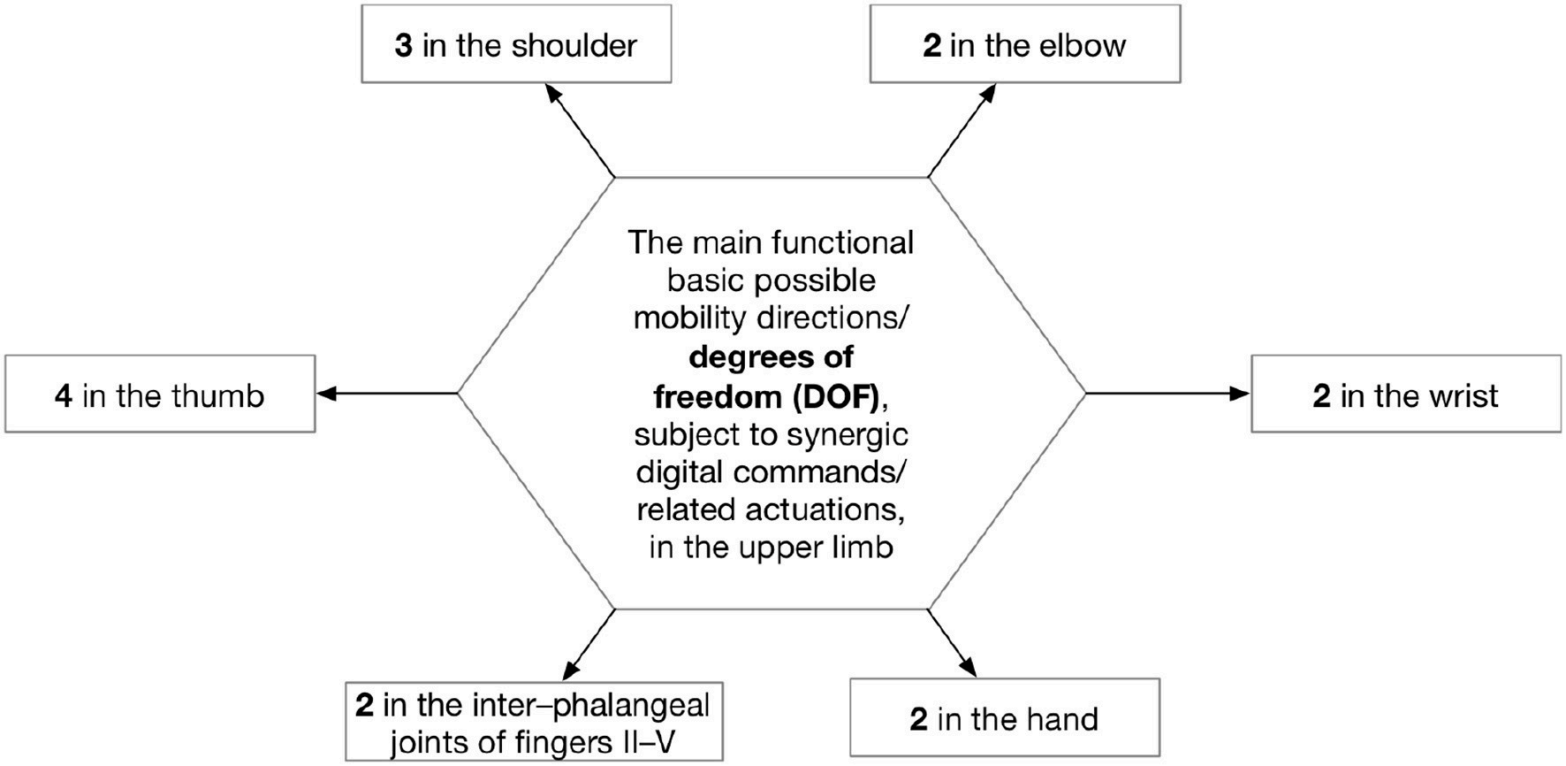
DOF for robot-assisted interventions in the upper limb (Sbenghe, 1987).

In order to improve the human-robot interaction and implicitly its effect, such devices should be more appropriate with the naturalistic/biomimetic kinematics of the upper limb following the main related degrees of freedom (DOF) (Figure 7).

#### 4.1.1. Potential beneficiary patients

The main pathology spectra in the upper limb, approachable by robotic devices evoked in the selected articles, consists of: stroke (the majority–41.66%) (Huang et al., 2012; Song et al., 2012; Kim et al., 2013; Wei et al., 2013; Xiao et al., 2014; Nycz et al., 2015; Kim and Rosen, 2015; Guo et al., 2016; Tu et al., 2017a,b), traumatic brain injury (Giberti et al., 2014), spinal cord injury (Miller and Rosen, 2010; Frisoli et al., 2016), Parkinson's disease/tremor (Rocon et al., 2007; Nimawat and Jailiya, 2015; Shull and Damian, 2015; Freer et al., 2017), peripheral nerve lesions in the upper limb–including with carpal tunnel syndrome–(Noveanu et al., 2013; Giberti et al., 2014; Andrikopoulos et al., 2015; Shull and Damian, 2015; Frisoli et al., 2016). Aside those above mentioned, in the additional literature we have studied, there are to be found also the following indications: Cerebral Palsy, Multiple Sclerosis, Spinal Muscular Atrophy, Brachial Plexus Injury, Arthrogryposis Multiplex Congenita (Rahman et al., 2006; Haumont et al., 2011; Maciejasz et al., 2014; Lopez et al., 2014; Gilliaux et al., 2015).

#### 4.1.2. Related targeted topography

“Taxonomy of these devices reflects the needs of different types of patients” (Onose et al., 2016). For instance, consider a severe (complete) paralysis of the whole upper limb [after stroke—within hemiplegia—or respectively, after a serious brachial plexus trauma, and additionally: even between different types of neural injuries that generate (morph–) functional deficit(s), there are assorted kinds of (adequate to the diagnosis) interventions]. This would demand the use of a more extended mechatronic, rehabilitative-assistive exoskeleton (as both: territory addressed—over the entire affected upper limb—and interfacing complexity needed/provided by such apparatus) than a distal lesion of the, for instance, ulnar nerve, which need such robotized orthotic interventions, basically, only over the hand region.

More detailed: conceptually analytic rehabilitation aims at (morph)–functional restoring of impairments, based on recovery and/or assistive targets (identified through clinical/para-clinical-recommended: quantitative or at least semi-quantitative-assessments). Most of the specific objectives, addressed by the assistive-rehabilitative endeavors (including) in the upper limb related pathology, basically result out of the upper motor neuron (UMN) and/or lower motor neuron (LMN) syndromes' semiological main items. In brief, these are, for the UMN syndrome: impairment/loss of voluntary movements and/or their coordination–motion force and speed and/or nicety decrease/weakness or abolishment (paresis or paralysis and/or respectively, apraxia/motor planning deficits)–, muscle hypertonia/spasticity, hyperreflexia/clonus (Reed, 2001; Basteris et al., 2014; Bryce, 2016) and for the LMN syndrome: impairment/loss of voluntary movements' control—motion force and speed decrease/weakness or abolishment (paresis or paralysis)—, hypo-/an-aesthesia, muscle hypotonia and atrophy (Purves et al., 2001; Bryce, 2016).

### 4.2. Main current technologies and categories of provided interventions

It is noteworthy to emphasize that mobile mechatronic/robotic orthoses/exoskeletons are—as expected with the related and general engineering advancements—also subject for actual and future construct, fabric and consequent performance, augmentations. Accordingly, such devices enable actuated controlled passive movements using VR or haptic capabilities (“wearables … untethered, ungrounded body worn devices that interact with skin directly or through clothing and can be used in natural environments outside a laboratory,” for “sensory replacement”/“augmentation” or training Shull and Damian, 2015). More interesting, for some of them, the haptic and VR facilities are coupled (Song and Guo, 2011; Huang et al., 2012; Song et al., 2013, 2014; Wei et al., 2013; Thielbar et al., 2014; Dowling et al., 2014; Grimm et al., 2016; Guo et al., 2016; Mazzoleni et al., 2017; Maris et al., 2018).

Another important technique is the electromyography (EMG)–feedbacks collected with skin surface electrodes, from the weakened muscles electrical signals. These serve, after filtered/processed into digital inputs, for the command of actuators capable to fulfill/amplify (in real-time adjustable manner) the voluntary—but otherwise impossible or impaired—movements in the “elbow/wrist/hand” (Bouzit et al., 2002; Kiguchi et al., 2008b; Stein, 2009; Frisoli et al., 2016). Additionally, it can be provided stimulation of the patient's own, biological actuators: his/her muscles, in the targeted anatomic region of the upper limb through functional electrical stimulation–FES–(Li et al., 2008) and/or, within the same paradigm, of pro-contractile mechanical input on muscle (tendons): vibratory stimulation (Lam et al., 2008; Shull and Damian, 2015).

The orthoses/exoskeletons “actuating” through FES, although not properly mechatronic–their power contribution to the respective segment's movements is not given through motors–can still be considered and discussed among the type of orthotic devices we focus on, because of their automated, robotic kind of interacting with the targeted area of upper limb with neuromotor impairment (Hu et al., 2015); the same goes for shape memory alloys (SMA–see further).

Regarding mobile devices, innovative systems are proposed in several articles (Yu and Rosen, 2010; Lenzi et al., 2011; Lin et al., 2013b; Chen et al., 2014; Giberti et al., 2014; Tageldeen et al., 2016; Xiao et al., 2017). The respective approaches differ by their mechanisms to generate rotational DOF:

- **Electric**: *using gear rings* (Chen et al., 2014) *cable transmission systems* (Xiao et al., 2017; Nycz et al., 2015); *moving cylinders* (Rocon et al., 2007; Lenzi et al., 2011; Wei et al., 2013).
- **Pneumatic**: (Balasubramanian et al., 2008; Yu and Rosen, 2010; Chen et al., 2014; Dowling et al., 2014; Lee, 2014; Andrikopoulos et al., 2015; Nycz et al., 2016; Tu et al., 2017a,b; Xiao et al., 2017) including *pneumatic muscle actuators* (Caldwell et al., 2007; Andrikopoulos et al., 2015).
- **Hydraulic/“hydropneumatic”** (Mistry et al., 2005; Noveanu et al., 2013; Lee, 2014; Polygerinos et al., 2015), including “*flexible fluidic actuators”* (Schill et al., 2011) or those using *electro and magneto rheological fluids (ERF-MRF)*.
- **“Shape memory alloys”–SMA–** (Rocon et al., 2007; Pittaccio et al., 2013, 2015; Kyrylova, 2015).

Aside the above described mechatronic infrastructure composition of the robotic orthoses/exoskeletons and their consequent actions interfering with the user, an equally important item underpinning the usefulness of such devices is the control loop closing, based on complex acquired inputs (Rocon et al., 2007; Yu and Rosen, 2010; Song and Guo, 2011; Song et al., 2012, 2013, 2014; Chen et al., 2014; Dowling et al., 2014; Andrikopoulos et al., 2015; Nimawat and Jailiya, 2015; Polygerinos et al., 2015; Nycz et al., 2016; Tageldeen et al., 2016):

- **Signals for controlling the device** (with the related sensoristics): measurement of interaction with the resistance opposed by the user and/or respectively, by the device and/or motion parameters (Giberti et al., 2014; Guo et al., 2016), including with spatial position of different segments of the upper limb/device (Powell and O'Malley, 2011).
- **Signals to trigger an action** (provided by different types of “switches”): manual commands and/or EMG/electroencephalography (EEG–non invasive brain computer/machine interface– BCI/BMI) and/or the contra-lateral, healthy limb movements and/or intervener forces between the user and the device (Ding et al., 2008; Kiguchi et al., 2008a; Marchal-Crespo and Reinkensmeyer, 2009; Guo et al., 2013; Basteris et al., 2014; Dowling et al., 2014; Maciejasz et al., 2014; Xiao et al., 2014; Frisoli et al., 2016; Tageldeen et al., 2016; Freer et al., 2017; Tu et al., 2017a,b).
- **Signals used to quantify the parameters' evolution** (mainly Range of Motion–ROM–, motor control, muscle strength and even, possibly, some current items regarding functionality–ADL type) give supplemental capability dimensions to modern more complex devices (Gilliaux et al., 2015; Maris et al., 2018).
- **VR capabilities** (Lin et al., 2013b) are another type of apparatus/software facility and consequent (mainly as adjunct) therapeutic rehabilitative method, especially higher motivating and credited as a moderate contributive treatment in improving rehabilitative outcomes (Lohse et al., 2014; Thielbar et al., 2014; Palma et al., 2017), some of them using ADL inspired “serious” gaming^12^ (Huang et al., 2012; Kim et al., 2013; Kim and Rosen, 2015; Frisoli et al., 2016; Tageldeen et al., 2016; Maris et al., 2018).

Newer and very challenging technological developments are expected to be implemented in the field we approached. If this would result in effectively mobile and completely functional, well tolerated, wearable such devices, this could represent a real breakthrough. Specifically, “soft body robots” (Nycz et al., 2015; Polygerinos et al., 2015) are bionic/biomimetic inspired, possibly actuated by “variable length tendons in the form of tension cables or shaped-memory alloy actuators” (as already afore exposed) or based on the expansion properties of elastomer structures, that can be powered either pneumatically or hydraulically (Dowling et al., 2014; Rus and Tolley, 2015; Polygerinos et al., 2015). Briefly resumed here, there are other two very important and useful features of such devices following the general trend of being more and more wearable and effective: anatomical/functional and or technical/structural modularity (Ding et al., 2008; Lo et al., 2010; Schill et al., 2011; Pearce et al., 2012; Noveanu et al., 2013; Lee, 2014; Xiao et al., 2014; Nycz et al., 2016) and complexity of “all-in-one”/hybrid kind (Giberti et al., 2014; Looned et al., 2014; Gandolla et al., 2017; Resquin et al., 2017; Tu et al., 2017a,b).

Other strong candidates for such a revolutionary advancement are: the (still) promising electroactive polymers (Rocon et al., 2007; Onose et al., 2016) and the (also still) near futuristic soft and stretchable electronics (Rus and Tolley, 2015). These may be suitable for incorporation within “sophisticated embodiments” if produced “…in microstructured and nanostructured forms, intimately integrated with elastomeric substrates …” - for instance: polydimethylsiloxane - and eventually maybe even resulting in “tissue-like” devices (Rogers et al., 2010).

**Figure 8:**
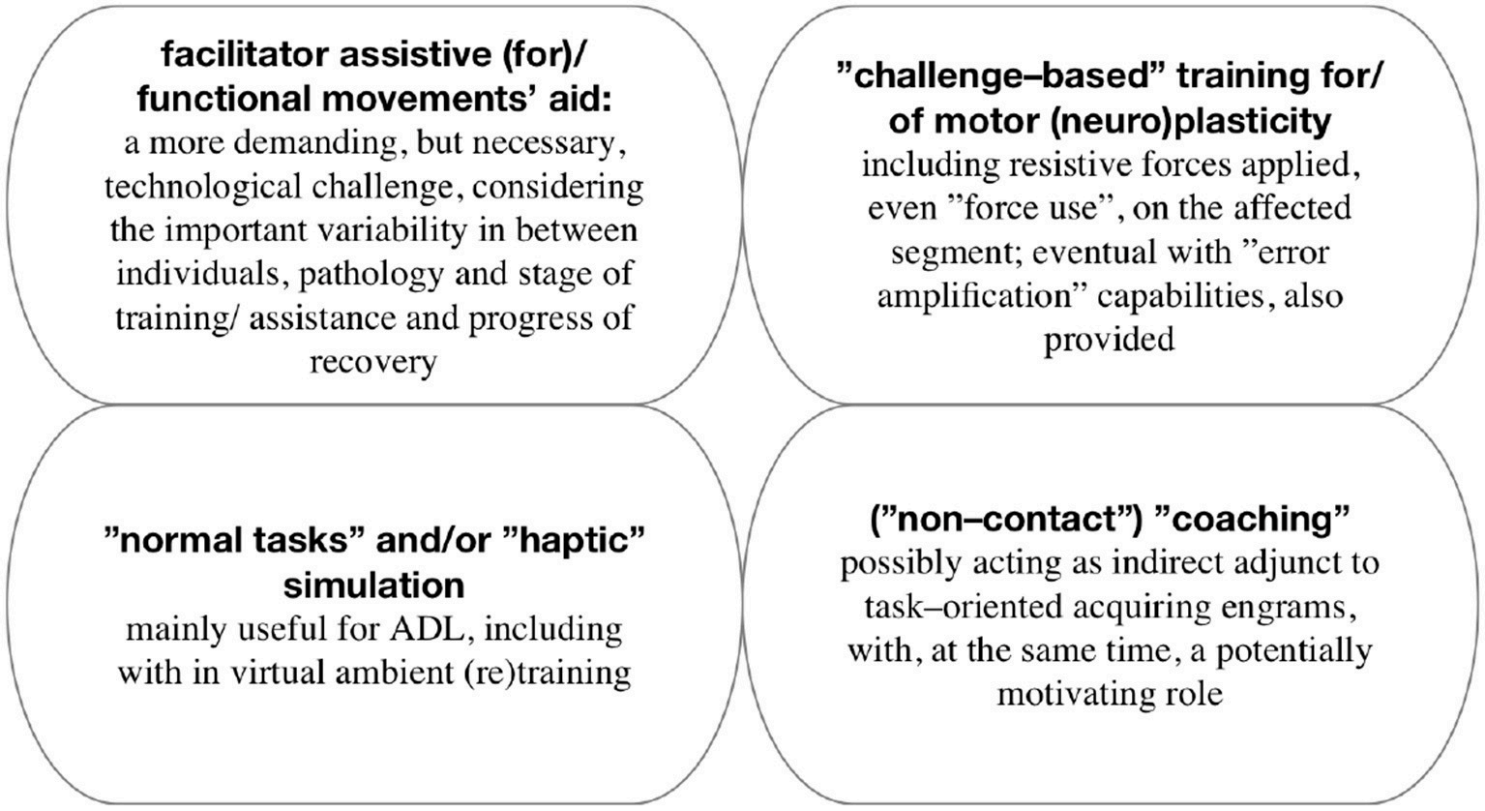
Taxonomy of main category interventions (Marchal-Crespo and Reinkensmeyer, 2009).

Regarding the **main categories of related interventions** provided, there are asserted as principal elements supporting “motor recovery,” the following: timely approach, adequate dosage, goal-settled exercise and patient's collaborative—if possible—involvement (Frisoli et al., 2016). Furthermore, these can be taxonomically systematized as shown in Figure 8.

### 4.3. Brief referring to main outcome assessment modalities

There doesn't seem to be a consensus in the literature about the consistency—both quantitative and qualitative—of the clinical research studies, deployed to date in the field (especially during the time frame considered, basically, by us, too—as afore specified). For example some authors consider that there are many studies using standardized scales (Marchal-Crespo and Reinkensmeyer, 2009). Others consider their number (including with related results) to be “sparse,” especially of clinical type (Lo, 2012; Maciejasz et al., 2014; Proietti et al., 2016). This partially explains the “slowly” growing recognition of the mechatronic-assisted therapies usefulness. But, however all of them acknowledge as being, numerically, on an ascendant trend.

To be noted that some of the measurement tools frequently used are–as known–comprehensive, being conceived to evaluate in detail, thoroughly the functioning (from the International Classification of Functioning, Disability and Health -ICF-perspective)^13^,^14^ aspects they are designated to quantify and hence, are very laborious and–inevitably–chronophagic. Thereby, the use of advanced, multimodal assistive-rehabilitative mechatronic/ robotic orthoses/ exoskeletons, provided including with (automated–so user friendly and investigators' time/ effort saver, and at the same time, standardized/ more prone to non-altered repeatability in “observational” and tracking use) assessment facilities, might represent a noteworthy alternative/ “Complement to Clinical Scales” (Mazzoleni et al., 2017).

In the 36 articles we have selected, there have been met the following assessment scales:

- Fugl–Meyer Assessment–FM(A)^15^ (APTA, 2011, pages: 51, 55–67, 70–74; Huang et al., 2012; Kim et al., 2013);
- Wolf Motor Function Test-WMFT (Taub et al., 2011; Huang et al., 2012; Takahashi et al., 2016);
- Active Range of Motion–AROM^16^ (Huang et al., 2012);
- Mini-Mental State Exam–MMSE^17^ (Kim et al., 2013);
- Peg-in-hole task^18^ (Miller and Rosen, 2010; Kim et al., 2012a,b; Kim and Rosen, 2015)-is a virtual or physical testing modality, resembling, in principle, to the standardized “9 Hole Peg Test” (APTA, 2011 page: 5).

In the supplementary literature we have studied, there have also been found—aside the above mentioned ones—around twenty assessment scales (at least about half of them very often mentioned/availed in different studies) (details in^19^).

## 5. Conclusions

According to the reasoning illustrated throughout this overview, the tenacious pursuing of basic—neuro-physiological, clinical and technological—but also translational research is warranted and we consider it has potential to reach the afore-mentioned therapeutic/rehabilitative and/or assistive goals. All these aim to improve the needing persons' overall functionality and consequent quality of life, additionally targeting, in the actual and future context, a more efficient skilled human resources use, within a globally improved related cases management approach paradigm. This is, at the same time, consistent with the fact that there are currently no available related spectacular bio-/pharmacological solutions for the central nervous system lesions' repair (Talley Watts et al., 2015).

Also important: as the industry of robotics with medical designations, including with the mobile—portable and/or wearable—orthoses/exoskeletons personally used to rehabilitate and/or assist neuromotor impairments in upper limbs, is progressing, “the regulatory science of powered exoskeletons is still developing.” Its utility regards such devices, too, despite the fact that, compared to the ones designated to be used in the lower limbs, these are safer, including: “free from the dangers associated with falling” (He et al., 2017). Therefore, although this might slow the advancement in the domain, more and more rigor is necessary and–on the long run–beneficial, in terms of safety for the users and emulator. At the same time, focus should also be maintained on a improving human-machine interaction through better technological and translational solutions.

Overall, to date, a certain multi-plane related diversity/“heterogeneity” (Basteris et al., 2014) can still be confirmed. This refers to: devices availed, trials carried out [as designs, participants (biometric and/or pathology data–and moreover, within each, on possible different evolution stages/respectively consequent onset of the robotic intervention(s), and/or severity degree of the neuromotor impairment at baseline), outcomes determined and evaluation methods used to measure them, different components of the administration dose, period(s) surveyed] and—partially—the related point wise and/or general conclusions on the subject matter, derived in various reviews/overviews, encompassing systematic ones, some of them with afferent meta-analyses fulfilled. Yet, this must not be so worrying, but rather motivating for perseverance, with constantly improved—mainly more unitary—research methodology. As long as the trend of growing interest for the domain of robotic assistive-rehabilitative approaches will continue, it will entail inherent—actual and very probable, future, too—controversies over different specific aspects; but more important, we reckon, it will leave the door open for progress as well.

## Author contributions

All authors listed have made a substantial, direct and intellectual contribution to the work, and approved it for publication.

### Conflict of interest statement

The authors declare that the research was conducted in the absence of any commercial or financial relationships that could be construed as a potential conflict of interest.

## Footnotes

^1^http://www.cochrane.org/

^2^https://www.ncbi.nlm.nih.gov

^3^https://www.ncbi.nlm.nih.gov

^4^https://www.elsevier.com/

^5^http://search.pedro.org.au/search

^6^https://apps.webofknowledge.com

^7^http://prisma-statement.org/documents/PRISMA%202009%20flow%20diagram.pdf

^8^Data, 2017, from: http://www.pedro.org.au/english/downloads/pedro-scale/

^9^Data, 2017, from: https://cris.maastrichtuniversity.nl/portal/files/549625/guid-964a90f5-d8aa-4c7f-9d74-b4b70552d067-ASSET1.0

^10^Data, 2017, from: https://www.strokengine.ca/glossary/pedro-score

^11^http://mjl.clarivate.com

^12^Individualized Technology and Robot-Assisted Virtual Learning Environment (I-TRAVLE) project– https://kenniscentrum.adelante-zorggroep.nl/en/research-programme/projects-2014/individualised-technology-and-robot-assisted-virtual-learning-environment-(i-travle)-www-i-travle-eu/

^13^Available online at: https://www.cdc.gov/nchs/data/icd/icfoverview_finalforwho10sept.pdf

^14^Available online at: http://apps.who.int/classifications/icfbrowser/

^15^http://www.neuropt.org/docs/edge-documents/stroke-edge-compendium-of-instructions2A86360E9D57.pdf?sfvrsn=2

^16^https://www.dshs.wa.gov/sites/default/files/FSA/forms/pdf/13-585a.pdf

^17^http://enotes.tripod.com/MMSE.pdf

^18^https://www.physio-pedia.com/Nine-Hole_Peg_Test

^19^https://cn.curs.pub.ro/scales.pdf
