# Supplementary material for: Mobile Mechatronic/Robotic Orthotic Devices to Assist–Rehabilitate Neuromotor Impairments in the Upper Limb: A Systematic and Synthetic Review
Source: Front Neurosci. 2018 Sep 5;12:577. doi: 10.3389/fnins.2018.00577 (PMC6134072; doi:10.3389/fnins.2018.00577)
Supplement: Supplementary file 1 [file Data_Sheet_1.pdf]

Supplementary Material: Mobile Mechatronic/ Robotic Orthotic Devices to Assist–Rehabilitate Neuromotor Impairments in the Upper Limb: A Systematic and Synthetic Review

APPENDIX 1 PEDRO INSPIRED SCORE FOR THE SELECTED ARTICLES – SORTED CHRONOLOGICALLY BY THEIR DATE OF PUBLICATION

No	Article	No of healthy subjects	No of patients	No of citations	Q1	Q2	Q3	Q4	PEDro score
1	Rocon et al. (2007)	0	10	203	5	5	5.0	3	9
2	Martinez et al. (2008)	1	0	30	3	2	0.3	2	4
3	Miller and Rosen (2010)	6	0	28	5	4	1.0	3	6
4	Yu and Rosen (2010)	0	0	47	5	1	1.5	5	6
5	Kim et al. (2012)	10	0	46	5	4	2.1	3	7
6	Huang et al. (2012)	11	22	21	5	5	0.4	2	6
7	Miller and Rosen (2010)	5	0	9	5	4	0.2	2	6
8	Pearce et al. (2012)	NA	NA	26	3	NA	1.2	5	6
9	Song et al. (2012)	1	0	11	5	2	0.2	3	5
10	Kim et al. (2013)	0	15	73	5	5	1.9	4	8
11	Lin et al. (2013a)	0	0	33	5	1	2.3	4	6
12	Guo et al. (2013)	0	0	13	5	1	1.2	2	5
13	Lin et al. (2013b)	0	0	0	5	1	0.5	1	4
14	Song et al. (2013)	1	0	2	5	2	0.6	4	6
15	Wei et al. (2013)	0	0	10	5	1	1.1	3	5
16	Noveanu et al. (2013)	NA	NA	4	5	NA	0.6	2	5
17	Giberti et al. (2014)	0	0	1	5	2	1.1	3	6
18	Song et al. (2014)	3	0	22	5	3	1.7	4	7
19	Dowling et al. (2014)	12	0	18	3	5	0.8	2	5
20	Xiao et al. (2014)	1	0	10	3	2	1.6	3	5
21	Andrikopoulos et al. (2015)	1	0	4	3	2	1.6	3	5
22	Nimawat and Jailiya (2015)	NA	NA	1	3	NA	1.5	2	4
23	Shull and Damian (2015)	NA	NA	38	5	NA	3.2	5	9
24	Nycz et al. (2015)	1	0	6	3	1	1.7	2	4
25	Kim and Rosen (2015)	10	0	7	5	4	2.1	5	8
26	Polygerinos et al. (2015)	1	0	214	5	2	5.0	5	9
27	Tageldeen et al. (2016)	0	0	1	3	1	2.1	3	5
28	Gao et al. (2016)	0	0	0	3	1	2.0	0	3
29	Nycz et al. (2016)	0	0	15	5	2	2.6	4	7

30	Guo et al. (2016)	0	0	0	5	1	2.0	3	6
31	Frisoli et al. (2016)	NA	NA	1	3	NA	2.1	3	5
32	Alavi et al. (2017)	1	0	0	5	2	2.5	4	7
33	Freer et al. (2017)	9	0	0	5	4	2.5	2	7
34	Gandolla et al. (2017)	3	0	0	3	3	2.5	2	5
35	Tu et al. (2017a)	3	0	0	5	3	2.5	4	7
36	Xiao et al. (2017)	0	0	0	5	1	2.5	2	5
37	Tu et al. (2017b)	1	0	0	5	2	2.5	4	7

Q1 – 5 points if indexed by Institute for Scientific Information (ISI) Thomson Reuters/ 3 points if indexed by other International Data Bases (IDB)

Q2 – number of citations per year pondered through the year of publication (described in section Methods of article's body text)

Q3* – number of human subjects included in the study, as follows:

**No. of human subjects**	**Q3 score**
0 healthy subjects/ 0 pacients	1
1 healthy subject/ 0 pacients	2
2–4 healthy subjects/ 1 – 2 pacients	3
5–10 healthy subjects/ 3 – 5 pacients	4
>10 healthy subjects/ >5 patients	5

Table S2. **Q3** score based on number of human subjects/ pacients

* This criterion does not apply to review articles: for such papers we considered the other three criteria, following the same calculation formula.

Q4 – the references' quality, as follows:

**No. of references**	**Q4 score**
0	0
1–10	1
11–20	2
21–30	3
31–40	4
$\geqslant 41$	5

Table S3. **Q4** score based on number of references

The total score of an article is obtained as the average of the points for each criterion multiplied by 2 (in order to range the maximal score up 10).

The PEDro inspired scoring primary data results have been statistically analyzed in table S4.

**Modal Value**	**5**
**Average**	**5.95**
**Median**	**6**
**Dispersion**	**1.81**
**Standard deviation**	**1.5**
**Coefficient of variation**	**25.23**
**Pearson asymmetry coefficient**	**0.63**

Table S4. Statistical analysis of the results

APPENDIX 2 MOBILE (WEARABLE AND/OR PORTABLE) PROTOTYPE DEVICES

Name and citation	Year	Brief description
The Motorized Upper-Limb Orthotic System (MULOS) (Johnson et al., 2001)	*2001*	*With 5 DOFs and attachable to a wheelchair, this prototype has multiple uses, including motorized assistance for severe disability, continuous passive motion and exercise device.*
Soft Robotic Exoskeleton (SRE) (Caldwell et al., 2007)	*2003*	*Weighing no more than 2 kg, this upper arm exoskeleton system has 7 DOFs by using rigid lightweight aluminium links combined with pneumatic muscle actuators.*
MAHI (Mechatronics and Haptic Interfaces lab) EXO-II (Gupta and O'Malley, 2006)	*2004*	*Having 4 active DOFs and 1 passive DOF, the robot is suitable for clinical usage by availing redundant safety measures, high accuracy quadrature encoders and reduced transmission rates. It is controlled by a computer running Simulink and Quark, thus reaching a command frequency of 1KHz.*
ASSIST (Sasaki et al., 2005)	*2005*	*Using two pneumatic soft actuators for palm and arm movement, this prototype targets elderly people/ those who need to be under care.*
Robotic Upper Extremity Repetitive Trainer/Therapy (RUPERT) IV (Kim et al., 2013; Tu et al., 2017a,b)	2005	RUPERT IV prototype has 5 actuated DOFs and uses iterative learning combined with a PID-based feedback controller in order to adapt to non-linear aspects generated by different patients performing various tasks.
Wearable Orthosis for Tremor Assessment and Suppression (WOTAS) (Rocon et al., 2007; Freer et al., 2017)	2007	Based on multiple types of sensors, including EMG, this prototype consists of a wearable orthosis designed to reduce tremor.
Muscle Assistant System (MAS) (Ding et al., 2008)	*2008*	*The prototype has a modular design combining posture measurement with muscle force estimation and power-assisting devices in a 4 DOFs structure. Unlike exoskeleton devices, the proposed prototype has no rigid frames and uses pneumatic actuators.*
Hybrid system (Varoto et al., 2008)	*2008*	*The elbow, wrist and hand prototype can be mounted on a wheelchair and is voice controlled directly by the patient*
Upper Extremity Exoskeleton (Moubarak et al., 2009)	*2009*	*Mounted on wheelchair, the proposed robot exoskeleton has 4 motorized DOFs commanded by a Dspace controller connected to Matlab/ Simulink using ControlDesk interface. In order to close the control loop, the device uses force sensors.*
Exoskeleton Robot (ExoRob) (Rahman et al., 2010)	2009	*A 2DOF wrist aluminium exoskeleton robot using DC motors that is worn on the lateral side and to help the pacient perform flexion/ extension*
NEUROExos (Vitiello et al., 2013)	2009	*This cable-driven elbow exoskeleton uses a remotely placed control unit equipped with two antagonist muscle-like hydraulic actuators*

Motion Assistive Exoskeleton-robot for Superior Extremity (ETS-MARSE) (Rahman et al., 2010)	*2010*	*Using a 7 DOFs exoskeleton, the proposed solution is controlled by using EMG signals in order to help patients in their daily routine.*
Upper-limb power-assist exoskeleton robot (Kiguchi et al., 2008)	*2010*	*Mounted on a wheelchair, the exoskeleton has 4-DOF power actuation and allows several rehabilitative motions such as: shoulder vertical/ horizontal flexion/ extension, elbow flexion/ extension and wrist supination/ pronation*
OrthoJacket (Schill et al., 2011)	*2010*	*Powered by hidraulic actuators, the "non-invasive modular hybrid neuro-orthosis" combines sensoritics with advanced signal processing techniques in order to identify patient intention and provide support*
euro Exos (Lenzi et al., 2011)	*2011*	*Includes a moving cylinder, micro compressor and controller. The torsion springs are mounted on the joints of each finger in order to provide assistive force. Each finger is driven by steel wire tendons which are supported by lightweight pulleys*
Upper limb's motion tracking exoskeleton device (Song and Guo, 2011)	*2011*	*Designed for home use, the 3 DOF exoskeleton device tracks the patient's movement by using an inertia sensor and is actuated by a DC motor.*
BMI-based occupational therapy assist suit (BOTAS) (Sakurada et al., 2013)	2013	*Even if it requires a computer connection in order to process the EEG signal, the device is fully wearable and uses a LED based system for signaling goal achievements (grasping and extension)*
Upper-limb exoskeleton rehabilitation device (ULERD) (Song et al., 2014)	2013	ULERD focuses on passive and resistance training by using 3 active DOFs and 4 passive DOFs controlled by a pulley system and three DC motors.
Wrist Gimbal (Martinez et al., 2013)	*2013*	*Using a simple arm rest with padding and straps, rubber hard stops on each axis and a simple design gives the 3 active DOFs exoskeleton prototype robustness and mechanical rigidity in a safe and practical manner for forearm and wrist rehabilitation.*
Upper limb exoskeleton (UL-EXO7) (Kim et al., 2013; Miller and Rosen, 2010; Yu and Rosen, 2010)	2013	This wearable 7 DOF Upper Limb Exoskeleton Robot uses a PID controlled articulation that enables a range-of-motion reaching 99%.
6DOF Robotic system (Noveanu et al., 2013)	2013	This 6DOF prototype introduces the usage of "smart fluids": electrorheological fluid (ERF) or magnetorheological fluid (MRF) for designing and implementing new robust braking systems.
Isolated Orthosis for Thumb Actuation (IOTA) (Aubin et al., 2013)	*2013*	*The prototype consistes of a 2 DOF thumb exoskeleton used in "pediatric at-home rehabilitation"*
Upper limb exoskeleton (Garrido et al., 2014)	*2014*	*The prototype has a modular structure and uses revolute joints in order to achieve the 4 DOFs required for arm rehabilitation*

6-DOF exoskeleton (Chen et al., 2014)	*2014*	*The prototype mechanism consists of multiple gear (straight and bevel) and support rings actuated through parallel joints in order to assist and analyze the patient's movement.*
Shoulder exoskeleton (Giberti et al., 2014)	2014	This hybrid structure characterized by a double parallel mechanism consists of a first platform fixed onto the body, near to the neck, a medium platform and a last platform. The actuators were chosen with a light weight in mind so that the whole system will not exceed 3 kg.
Soft robotic glove (Polygerinos et al., 2015)	2014	This orthotic device is actuated by using a hydraulic muscle like cylinders in order to perform several rehabilitation movements placed on a waist belt.
BCI-driven exoskeleton (Xiao et al., 2014)	2014	The 4 DOF exoskeleton is controlled by using a EEG based device (BCI) in order to assist the patient.
Wearable exoskeleton robotic hand/arm (Lee, 2014)	*2014*	*The 9 DOF robotic hand/arm exoskeleton is ultra light (300g) and combines electrical with mechanical (springs) actuators.*
Robotic Arm Orthosis (RAO) (Looned et al., 2014)	*2014*	*The system consists of a wearable hybrid prototype combining an elbow, wrist and hand robotic exoskeleton device with FES and BCI.*
Six-degrees-of-freedom upper-limb exoskeleton robot (6-REXOS) (Gunasekara et al., 2015)	*2015*	*The prototype focuses on improving the pHRI (physical human-robot interaction) by using 4 active rotational DOFs and 2 passive translational DOFs, thus ensuring movement redundancy and reduced misalignments.*
EXOskeletal WRIST (EXOWRIST) (Andrikopoulos et al., 2015)	2015	Involves pneumatic muscle actuators. This stands for the reliability and portability of robust robotic solutions for wrist rehabilitation. This approach enables two DOFs movements to be performed: extension/ flexion and ulnarradial deviation. It can be successfully integrated into a lot of rehabilitation exoskeletal concepts for the assisted movement of the upper limb
Soft glove and sleeve (Nycz et al., 2015)	2015	The prototype is constructed as a soft glove and sleeve that assists the patient's finger and elbow movements by using tendon-like actuated cables.
EMG-based upper limb exoskeleton (Tageldeen et al., 2016)	2016	The centerpiece of this prototype is a fuzzy controller based on torque estimation techniques. By providing an interactive gaming software environment, this prototype assists and engages patients in order to increase the rehabilitation efficiency.
Cable-driven upper limb exoskeleton (CABexo) (Xiao et al., 2017)	2017	The 6-DOF wearable system uses a complex structure of epicyclic gear trains controlled by cables.
BRIDGE exoskeleton (Gandolla et al., 2017)	2017	The light wearable exoskeleton has 5 DOFs controlled directly by the patient and is an extension of the passive MUNDUS exoskeleton that can be mounted on wheelchairs.

REFERENCES

Alavi, N., Zampierin, S., Komeili, M., Cocuzza, S., Debei, S., and Menon, C. (2017). A preliminary investigation into the design of pressure cushions and their potential applications for forearm robotic orthoses. *BioMedical Engineering OnLine* 16, 54. doi:10.1186/s12938-017-0345-8

Andrikopoulos, G., Nikolakopoulos, G., and Manesis, S. (2015). Motion control of a novel robotic wrist exoskeleton via pneumatic muscle actuators. In *2015 IEEE 20th Conference on Emerging Technologies Factory Automation (ETFA)*. 1–8. doi:10.1109/ETFA.2015.7301464

Aubin, P. M., Sallum, H., Walsh, C., Stirling, L., and Correia, A. (2013). A pediatric robotic thumb exoskeleton for at-home rehabilitation: the Isolated Orthosis for Thumb Actuation (IOTA). *IEEE Int Conf Rehabil Robot* 2013, 6650500. [DOI:10.1109/ICORR.2013.6650500] [PubMed:24187315]

Caldwell, D. G., Tsagarakis, N. G., Kousidou, S., Costa, N., and Sarakoglu, I. (2007). Soft exosckeletons for upper and lower body rehabilitation - design, control and testing. *International Journal of Humanoid Robotics* 04, 549–573. doi:10.1142/S0219843607001151

Chen, Y., Li, G., Zhu, Y., Zhao, J., and Cai, H. (2014). Design of a 6-DOF upper limb rehabilitation exoskeleton with parallel actuated joints. *Biomed Mater Eng* 24, 2527–2535. [DOI:10.3233/BME-141067] [PubMed:25226954]

Ding, M., Ueda, J., and Ogasawara, T. (2008). Pinpointed muscle force control using a power-assisting device: System configuration and experiment. In *2008 2nd IEEE RAS EMBS International Conference on Biomedical Robotics and Biomechatronics*. 181–186. doi:10.1109/BIOROB.2008.4762829

Dowling, A. V., Barzilay, O., Lombrozo, Y., and Wolf, A. (2014). An Adaptive Home-Use Robotic Rehabilitation System for the Upper Body. *IEEE J Transl Eng Health Med* 2, 2100310. [PubMed Central:PMC4848105] [DOI:10.1109/JTEHM.2014.2314097] [PubMed:27170877]

Freer, D. R., Liu, J., and Yang, G. Z. (2017). Optimization of emg movement recognition for use in an upper limb wearable robot. In *2017 IEEE 14th International Conference on Wearable and Implantable Body Sensor Networks (BSN)*. 202–205. doi:10.1109/BSN.2017.7936041

Frisoli, A., Solazzi, M., Loconsole, C., and Barsotti, M. (2016). New generation emerging technologies for neurorehabilitation and motor assistance. *Acta Myol* 35, 141–144. [PubMed Central:PMC5416742] [PubMed:28484314]

Gandolla, M., Costa, A., Aquilante, L., Gfoehler, M., Puchinger, M., Braghin, F., et al. (2017). Bridge - behavioural reaching interfaces during daily antigravity activities through upper limb exoskeleton: Preliminary results. In *2017 International Conference on Rehabilitation Robotics (ICORR)*. 1007–1012. doi:10.1109/ICORR.2017.8009381

Gao, F., Li, G., Wu, H., Wang, Q., Liu, J., and Keogh, J. (2016). Advances in Rehabilitation and Assistive Robots for Restoring Limb Function in Persons with Movement Disorders. *Appl Bionics Biomech* 2016, 3864728. [PubMed Central:PMC4992748] [DOI:10.1155/2016/3864728] [PubMed:27578958]

Garrido, J., Yu, W., and Soria, A. (2014). Modular design and modeling of an upper limb exoskeleton. In *5th IEEE RAS/EMBS International Conference on Biomedical Robotics and Biomechatronics*. 508–513. doi:10.1109/BIOROB.2014.6913828

Giberti, H., Bertoni, V., and Coppola, G. (2014). Conceptual design and feasibility study of a novel upper-limb exoskeleton. In *2014 IEEE/ASME 10th International Conference on Mechatronic and Embedded Systems and Applications (MESA)*. 1–6. doi:10.1109/MESA.2014.6935548

Gunasekara, M., Gopura, R., and Jayawardena, S. (2015). 6-rexos: Upper limb exoskeleton robot with improved phri. *International Journal of Advanced Robotic Systems* 12, 47. doi:10.5772/60440

Guo, S., Gao, J., Guo, J., Zhang, W., and Hu, Y. (2016). Design of the structural optimization for the upper limb rehabilitation robot. In *2016 IEEE International Conference on Mechatronics and Automation*. 1185–1190. doi:10.1109/ICMA.2016.7558730

Guo, S., Zhang, F., Wei, W., Guo, J., and Ge, W. (2013). Development of force analysis-based exoskeleton for the upper limb rehabilitation system. In *2013 ICME International Conference on Complex Medical Engineering*. 285–289. doi:10.1109/ICCME.2013.6548256

Gupta, A. and O'Malley, M. K. (2006). Design of a haptic arm exoskeleton for training and rehabilitation. *IEEE/ASME Transactions on Mechatronics* 11, 280–289. doi:10.1109/TMECH.2006.875558

Huang, S., Luo, C., Ye, S., Liu, F., Xie, B., Wang, C., et al. (2012). Motor impairment evaluation for upper limb in stroke patients on the basis of a microsensor. *Int J Rehabil Res* 35, 161–169. [DOI:10.1097/MRR.0b013e328353053a] [PubMed:22470053]

Johnson, G. R., Carus, D. A., Parrini, G., Scattareggia Marchese, S., and Valeggi, R. (2001). The design of a five-degree-of-freedom powered orthosis for the upper limb. *Proc Inst Mech Eng H* 215, 275–284. [DOI:10.1243/0954411011535867] [PubMed:11436270]

Kiguchi, K., Liyanage, M., and Kose, Y. (2008). Intelligent perception assist with optimum force vector modification for an upper-limb power-assist exoskeleton. In *2008 2nd IEEE RAS EMBS International Conference on Biomedical Robotics and Biomechatronics*. 175–180. doi:10.1109/BIOROB.2008.4762790

Kim, H., Miller, L. M., Byl, N., Abrams, G. M., and Rosen, J. (2012). Redundancy resolution of the human arm and an upper limb exoskeleton. *IEEE Transactions on Biomedical Engineering* 59, 1770–1779. doi:10.1109/TBME.2012.2194489

Kim, H., Miller, L. M., Fedulow, I., Simkins, M., Abrams, G. M., Byl, N., et al. (2013). Kinematic data analysis for post-stroke patients following bilateral versus unilateral rehabilitation with an upper limb wearable robotic system. *IEEE Trans Neural Syst Rehabil Eng* 21, 153–164. [DOI:10.1109/TNSRE.2012.2207462] [PubMed:22855233]

Kim, H. and Rosen, J. (2015). Predicting redundancy of a 7 dof upper limb exoskeleton toward improved transparency between human and robot. *J. Intell. Robotics Syst.* 80, 99–119. doi:10.1007/s10846-015-0212-4

Lee, Y. K. (2014). Design of exoskeleton robotic hand/arm system for upper limbs rehabilitation considering mobility and portability. In *2014 11th International Conference on Ubiquitous Robots and Ambient Intelligence (URAI)*. 540–544. doi:10.1109/URAI.2014.7057385

Lenzi, T., Vitiello, N., Rossi, S. M. M. D., Persichetti, A., Giovacchini, F., Roccella, S., et al. (2011). Measuring human-robot interaction on wearable robots: A distributed approach. *Mechatronics* 21, 1123 – 1131. doi:https://doi.org/10.1016/j.mechatronics.2011.04.003

Lin, P.-Y., Shieh, W.-B., and Chen, D.-Z. (2013a). A theoretical study of weight-balanced mechanisms for design of spring assistive mobile arm support (mas). *Mechanism and Machine Theory* 61, 156 – 167. doi:https://doi.org/10.1016/j.mechmachtheory.2012.11.003

Lin, Y.-C., Jog, S., and Chang, J.-Y. J. (2013b). Pose estimation and simulation of upper limb exoskeleton. *ASME 2013 Conference on Information Storage and Processing Systems*

Looned, R., Webb, J., Xiao, Z. G., and Menon, C. (2014). Assisting drinking with an affordable bci-controlled wearable robot and electrical stimulation: a preliminary investigation. *Journal of NeuroEngineering and Rehabilitation* 11, 51. doi:10.1186/1743-0003-11-51

Martinez, F., Retolaza, I., Pujana-Arrese, A., Cenitagoya, A., Basurko, J., and Landaluze, J. (2008). Design of a five actuated dof upper limb exoskeleton oriented to workplace help. In *2008 2nd IEEE RAS*

EMBS International Conference on Biomedical Robotics and Biomechatronics. 169–174. doi:10.1109/BIOROB.2008.4762788

Martinez, J. A., Ng, P., Lu, S., Campagna, M. S., and Celik, O. (2013). Design of Wrist Gimbal: a forearm and wrist exoskeleton for stroke rehabilitation. *IEEE Int Conf Rehabil Robot* 2013, 6650459. [DOI:10.1109/ICORR.2013.6650459] [PubMed:24187276]

Miller, L. M. and Rosen, J. (2010). Comparison of multi-sensor admittance control in joint space and task space for a seven degree of freedom upper limb exoskeleton. In *2010 3rd IEEE RAS EMBS International Conference on Biomedical Robotics and Biomechatronics*. 70–75. doi:10.1109/BIOROB.2010.5628069

Moubarak, S., Pham, M., Pajdla, T., and T., R. (2009). Design and modeling of an upper extremity exoskeleton. In *World Congress on Medical Physics and Biomedical Engineering*, ed. Springer

Nimawat, D. and Jailiya, P. R. S. (2015). Requirement of wearable robots in current scenario. *European Journal of Advances in Engineering and Technology* 2, 19–23

Noveanu, S., Chetran, B., Tatar, O., Raducanu, G., and Mândru, D. (2013). Structural synthesis of the upper limb modular wearable exercisers. In *2013 17th International Conference on System Theory, Control and Computing (ICSTCC)*. 693–698. doi:10.1109/ICSTCC.2013.6689041

Nycz, C. J., Bützer, T., Lambercy, O., Arata, J., Fischer, G. S., and Gassert, R. (2016). Design and characterization of a lightweight and fully portable remote actuation system for use with a hand exoskeleton. *IEEE Robotics and Automation Letters* 1, 976–983. doi:10.1109/LRA.2016.2528296

Nycz, C. J., Delph, M. A., and Fischer, G. S. (2015). Modeling and design of a tendon actuated soft robotic exoskeleton for hemiparetic upper limb rehabilitation. In *2015 37th Annual International Conference of the IEEE Engineering in Medicine and Biology Society (EMBC)*. 3889–3892. doi:10.1109/EMBC.2015.7319243

Pearce, A. J., Adair, B., Miller, K., Ozanne, E., Said, C., Santamaria, N., et al. (2012). Robotics to enable older adults to remain living at home. *Journal of Aging Research* 2012, 10 pages

Polygerinos, P., Wang, Z., Galloway, K. C., Wood, R. J., and Walsh, C. J. (2015). Soft robotic glove for combined assistance and at-home rehabilitation. *Robot. Auton. Syst.* 73, 135–143. doi:10.1016/j.robot.2014.08.014

Rahman, M. H., Ouimet, T. K., Saad, M., Kenné, J. P., and Archambault, P. S. (2010). Development and control of a wearable robot for rehabilitation of elbow and shoulder joint movements. In *IECON 2010 - 36th Annual Conference on IEEE Industrial Electronics Society*. 1506–1511. doi:10.1109/IECON.2010.5675459

Rocon, E., Belda-Lois, J. M., Ruiz, A. F., Manto, M., Moreno, J. C., and Pons, J. L. (2007). Design and validation of a rehabilitation robotic exoskeleton for tremor assessment and suppression. *IEEE Transactions on Neural Systems and Rehabilitation Engineering* 15, 367–378. doi:10.1109/TNSRE.2007.903917

Sakurada, T., Kawase, T., Takano, K., Komatsu, T., and Kansaku, K. (2013). A BMI-based occupational therapy assist suit: asynchronous control by SSVEP. *Front Neurosci* 7, 172. [PubMed Central:PMC3779864] [DOI:10.3389/fnins.2013.00172] [PubMed:24068982]

Sasaki, D., Noritsugu, T., and Takaiwa, M. (2005). Development of active support splint driven by pneumatic soft actuator (assist). In *Proceedings of the 2005 IEEE International Conference on Robotics and Automation*. 520–525. doi:10.1109/ROBOT.2005.1570171

Schill, O., Wiegand, R., Schmitz, B., Matthies, R., Eck, U., Pylatiuk, C., et al. (2011). OrthoJacket: an active FES-hybrid orthosis for the paralysed upper extremity. *Biomed Tech (Berl)* 56, 35–44. [DOI:10.1515/BMT.2010.056] [PubMed:21210758]

Shull, P. B. and Damian, D. D. (2015). Haptic wearables as sensory replacement, sensory augmentation and trainer – a review. *Journal of NeuroEngineering and Rehabilitation* 12, 59. doi:10.1186/s12984-015-0055-z

Song, Z. and Guo, S. (2011). Development of a real-time upper limb's motion tracking exoskeleton device for active rehabilitation using an inertia sensor. In *2011 9th World Congress on Intelligent Control and Automation*. 1206–1211. doi:10.1109/WCICA.2011.5970708

Song, Z., Guo, S., Pang, M., Zhang, S., Xiao, N., Gao, B., et al. (2014). Implementation of resistance training using an upper-limb exoskeleton rehabilitation device for elbow joint. *Journal of Medical and Biological Engineering* 34, 188–196

Song, Z., Guo, S., Xiao, N., Gao, B., and Shi, L. (2012). Implementation of human-machine synchronization control for active rehabilitation using an inertia sensor. *Sensors (Basel)* 12, 16046–16059. [PubMed Central:PMC3571770] [DOI:10.3390/s121216046] [PubMed:23443366]

Song, Z., Wang, Z., Guo, S., and Gao, B. (2013). Study on resistance training for upper-limb rehabilitation using an exoskeleton device. In *2013 IEEE International Conference on Mechatronics and Automation*. 932–938. doi:10.1109/ICMA.2013.6618040

Tageldeen, M. K., Elamvazuthi, I., and Perumal, N. (2016). Motion control for a multiple input rehabilitation wearable exoskeleton using fuzzy logic and pid. In *2016 IEEE 14th International Workshop on Advanced Motion Control (AMC)*. 473–478. doi:10.1109/AMC.2016.7496395

Tu, X., Han, H., Huang, J., Li, J., Su, C., Jiang, X., et al. (2017a). Upper Limb Rehabilitation Robot Powered by PAMs Cooperates with FES Arrays to Realize Reach-to-Grasp Trainings. *J Healthc Eng* 2017, 15 pages. [DOI:10.1155/2017/1282934] [PubMed:29068628]

Tu, X., Zhou, X., Li, J., Su, C., Sun, X., Han, H., et al. (2017b). Iterative learning control applied to a hybrid rehabilitation exoskeleton system powered by pam and fes. *Cluster Computing* 20, 2855–2868. doi:10.1007/s10586-017-0880-x

Varoto, R., Barbarini, E. S., and Cliquet, A. (2008). A hybrid system for upper limb movement restoration in quadriplegics. *Artif Organs* 32, 725–729. [DOI:10.1111/j.1525-1594.2008.00597.x] [PubMed:18684204]

Vitiello, N., Lenzi, T., Roccella, S., Rossi, S. M. M. D., Cattin, E., Giovacchini, F., et al. (2013). Neuroexos: A powered elbow exoskeleton for physical rehabilitation. *IEEE Transactions on Robotics* 29, 220–235. doi:10.1109/TRO.2012.2211492

Wei, W., Guo, S., Zhang, F., Guo, J., Ji, Y., and Wang, Y. (2013). A novel upper limb rehabilitation system with hand exoskeleton mechanism. In *2013 IEEE International Conference on Mechatronics and Automation*. 285–290. doi:10.1109/ICMA.2013.6617932

Xiao, F., Gao, Y., Wang, Y., Zhu, Y., and Zhao, J. (2017). Design of a wearable cable-driven upper limb exoskeleton based on epicyclic gear trains structure. *Technol Health Care* 25, 3–11. [DOI:10.3233/THC-171300] [PubMed:28582886]

Xiao, Z. G., Elnady, A. M., Webb, J., and Menon, C. (2014). Towards a brain computer interface driven exoskeleton for upper extremity rehabilitation. In *5th IEEE RAS/EMBS International Conference on Biomedical Robotics and Biomechatronics*. 432–437. doi:10.1109/BIOROB.2014.6913815

Yu, W. and Rosen, J. (2010). A novel linear pid controller for an upper limb exoskeleton. In *49th IEEE Conference on Decision and Control (CDC)*. 3548–3553. doi:10.1109/CDC.2010.5716985
